# Kratom Use in the US: Both a Regional Phenomenon and a White Middle-Class Phenomenon? Evidence From NSDUH 2019 and an Online Convenience Sample

**DOI:** 10.3389/fphar.2021.789075

**Published:** 2021-12-20

**Authors:** Jeffrey M. Rogers, Kirsten E. Smith, Justin C. Strickland, David H. Epstein

**Affiliations:** ^1^ Real-world Assessment, Prediction, and Treatment Unit, National Institute on Drug Abuse Intramural Research Program, Baltimore, MD, United States; ^2^ Department of Psychiatry and Behavioral Sciences, Johns Hopkins University School of Medicine, Baltimore, MD, United States

**Keywords:** rural drug use, substance use disorder treatment, substance use disorder, opioids, mitragyna speciosa, kratom

## Abstract

Kratom products available in the United States are becoming increasingly diverse both in terms of content and in terms of how they are marketed. Prior survey research indicates that kratom has been primarily used in the US to self-treat anxiety, depression, pain, fatigue, and substance use disorder (SUD) symptoms. Kratom is also well-known for its use as a short- or long-term full opioid agonist substitute. Therefore, use may be greater in regions particularly impacted by addiction to prescription opioids. Use may also be greater in demographic groups targeted by media outlets (such as specific podcasts) in which kratom is touted. Here, we aimed to determine whether lifetime and past-year kratom use were associated with region of residence and with being young, White, post-secondary educated, and employed. To strengthen confidence in our findings, we analyzed data from two sources: our own crowdsourced online convenience sample and the 2019 National Survey on Drug Use and Health (NSDUH). In our sample (N = 2,615), 11.1% reported lifetime and 6.7% reported past-year kratom use, and the odds of kratom use were higher among people who were White, younger, at least high school educated, employed, and above the poverty line, as well as those reporting nonmedical opioid use, past-year SUD, or lifetime SUD treatment; residence was not a significant predictor. In NSDUH data, suburban residence and other demographic factors, concordant with those from the crowdsourced sample, were associated with kratom use. Taken together, the findings support a general “White middle-class suburban” profile of the modal kratom user, but more research is needed to understand it. In the interim, focus should be on our finding that lifetime nonmedical opioid use was associated with an up to five times greater likelihood of past-year kratom use, suggesting that drug-use history may presently be the strongest predictor of kratom use.

## Introduction

Kratom, the lay term referring to the Mitragyna speciosa Korth [Rubiaceae] tree native to Southeast Asia, has leaves that contain at least over 40 alkaloids with pharmacologic activity. Most notable among these with dose-dependent psychoactive effects are mitragynine (MG) and 7-hydroxymitragynine (7-HMG). Both alkaloids bind to and partially agonize the mu-opioid receptor, producing analgesic, stimulatory, and anxiolytic effects (Kruegel and Grundmann, 2018; Kruegel et al., 2019; Obeng et al., 2021; Todd, et al., 2020). While some of these effects can likely be attributed to mu-opioid receptor activity, others may occur through separate mechanisms (Hiranita et al., 2019).

Although use of the kratom leaf in Southeast Asia dates back at least to the early 1800s (Jansen and Prast, 1988), kratom use was not generally noted in the United States (US) until the early-mid 2000s (Boyer et al., 2007; Babu et al., 2008) and did not become widespread until approximately 2015 (Grundmann, 2017; Smith and Lawson, 2017). Currently, a variety of kratom products (loose leaf, powder, capsules, concentrate) can be legally purchased from online retailers, smoke shops, convenience stores, and specialty supplement shops in 46 US states (Griffin et al., 2016; Fowble and Musah, 2019). Exploratory surveys in the US seeking to better understand kratom use, motivations, and effects have found that many people report using kratom to “self-manage” chronic pain, fatigue, psychiatric, and symptoms of substance use disorders (SUDs), including opioid-withdrawal symptom relief and/or as a replacement for full opioid agonists (Bath et al., 2020; Boyer et al., 2008; Coe, et al., 2019; Garcia-Romeu, et al., 2020; Grundmann, 2017; Smith and Lawson, 2017; Swogger and Walsh, 2018).

### Kratom’s Relevance to Rural Regions

These latter two opioid-related motivations for use indicate that kratom use in the US may vary by region. Kratom’s relevance to people’s needs (and thus its prevalence of use) may be greater in rural communities that experienced higher per capita rates of opioid prescribing during the early 2000s and subsequently experienced changes in the licit and illicit prescription opioid market (Thomas et al., 2020). Findings consistently indicate high opioid-related risk for those living in rural settings: opioid prescribing is up to 33% higher in rural counties than elsewhere; rural-residing adolescents are more likely than those in urban-metro counties to initiate nonmedical use of opioids; rural justice-involvement carries a five-fold greater likelihood of nonmedical use of opioids; and overdose death rates for nonmedical use of opioids are 20–30% higher in rural counties (Havens et al., 2007; Paulozzi and Xi, 2008; Havens et al., 2011; Mack et al., 2017; Mosher et al., 2017; Ayres and Jalal, 2018; Luu et al., 2019). These outcomes are compounded by the practical and social difficulties of accessing treatment for opioid use disorder (OUD) in rural counties, including stigma surrounding medication for OUD (MOUD) (Bunting et al., 2018; Jones, 2018; Jacobson et al., 2020; Lister et al., 2020; Cole et al., 2021; Franz et al., 2021). Recent findings suggest that only half of physicians authorized to prescribe MOUD had the availability to accept new patients (Andrilla et al., 2018), and though MOUD access is increasing nationally and gains have been made to increase prescriber capacity in underserved areas (Barnett et al., 2019), more than half of small and rural counties lack a physician waivered by the Drug Enforcement Administration to prescribe MOUD (Andrilla and Patterson, 2021). Given the high prevalence of prescription opioid misuse, poor psychiatric (Snell-Rood and Carpenter-Song, 2018) and physical health (including high rates of chronic pain) (Meit et al., 2017), and the difficulty in obtaining MOUD in rural areas (Sexton et al., 2008; Prunuske et al., 2014; Woolf et al., 2019; Monnat, 2020), it is possible that kratom use might be more prevalent in rural counties than in urban-metro counties. Although heroin use is increasing in many rural communities that had elevated rates of opioid prescribing (Nolte et al., 2020; Schnell et al., 2020; Hedegaard and Spencer, 2021; Strickland et al., 2021), kratom might be more accessible or more attractive than heroin to people whose sole prior opioid use had involved prescribable pills. To date, kratom use has not been well characterized in terms of rural/suburban/urban differences. Only two large US survey studies have noted the geographic region of kratom users in their sample, both finding that a slightly greater proportion resided in the US South (Coe et al., 2019; Garcia-Romeu et al., 2020). However, in separate analyses, Nicewonder et al. (2019) found that kratom use was more widely distributed across the US, with higher rates in Florida, as well as Oregon, California, and Idaho, and still noteworthy use in the Northeast. These findings were from data collected in 2017; given the relatively recency of kratom’s emergence in the US, an update would probably be informative.

### But is Kratom Use More Than Self-Treatment and Opioid Replacement?

As kratom popularity in the US has grown substantially, there may be new subpopulations of kratom users that are distinct from those using kratom to address pain, psychiatric symptoms, and/or SUDs. In our own analyses of social-media posts, we found that some people are using kratom not to “self-treat” symptoms but rather to enhance mood and performance and to boost energy (Smith et al., 2021a; Smith et al., 2021b).

Using articles and books published in popular media outlets as a proxy, we can observe that kratom is now being advertised and sought out as a performance-boosting (“nootropic”) or wellness supplement (Mun and Wong, 2020; Carcache de Blanco and Kinghorn, 2021; Ng et al., 2021). A recent content analysis of over 42,000 comments made on kratom-related YouTube videos found that 50% reported use of kratom for its energy-boosting effects and 25% for its purported nootropic effects (Prevete et al., 2021). Though these motivations do not seem to represent a majority of kratom-using people, interest in kratom as a nootropic could expand interest in kratom and increase purchasing and use for groups other than those seeking to self-manage health conditions. For such groups, kratom products would likely be conceptualized as a wellness or performance-enhancing supplement, not a medication to alleviate underlying health symptoms. Indicative of expanding interest and popularization, discussions about kratom and its effects have been featured on popular media outlets such as The Joe Rogan Experience, one of the most downloaded podcasts in 2019 and 2020, with individual episodes garnishing up to 45 million views on YouTube alone (Rogan, 2018; Rogan, 2019; Jarvey, 2020). Precise figures on the demographics of podcast listenership are not readily available, but one informal survey estimates that The Joe Rogan Experience listenership is 24 years of age on average, 71% male, 50% post-secondary educated, and fairly high in income (76% reported earning over $50,000 USD annually). The male skew appears to be driven mostly by trends in overall podcast listenership, as equal proportions of podcast-listening men and women reported listening to The Joe Rogan Experience (Media Monitors, 2021). From these findings, we may expect to see greater likelihood of kratom initiation among people who constitute the demographic being more frequently exposed to promotion of kratom in specific types of content—people who are young, White, post-secondary educated, and employed.

Some evidence from national surveys does suggest greater kratom use prevalence among White, educated men, although the findings are mixed. A national-level convenience sample of over 8,000 kratom users was majority non-Hispanic White (89%) and male (57%), with at least some college-educated (82%), and earning annual incomes exceeding $35,000 USD (Grundmann, 2017). More recent nationally representative data from the Cross-sectional Survey of Non-Medical Use of Prescription Drugs (NMURx) Program 2018 – 2019 found that kratom use was not associated with income or race/ethnicity but was represented by a male majority (Schimmel et al., 2021). Meanwhile, data from the nationally representative National Survey on Drug Use and Health (NSDUH) in 2019 suggest decreased odds for past-year kratom consumption among people of Hispanic and Black race/ethnicity compared with White people, but found no robust associations with education level or annual family income (Palamar, 2021).

### Aims

We sought to address each of the two demographic considerations just discussed: whether kratom use is associated with rurality (versus urbanicity), and, in parallel, whether there is also an emerging culture of kratom use (possibly for different reasons, though we did not address that here) among people who are young, White, post-secondary educated, and employed. We used two independent data sources: our own crowdsourced online convenience sample of people reporting past 6 month alcohol, opioid, and/or stimulant use, and the 2019 National Survey on Drug Use and Health (NSDUH). By examining data from two distinct surveys with divergent sampling and assessment methods we hoped to find some convergence in results. Still, we did not have a priori hypotheses as to whether we would find such convergence, or even whether indirect evidence of a kratom-user typology, characteristic of the one described above, would be found.

## Methods

This secondary data analyses examined responses from two different US-based surveys, neither of which sought to recruit based on kratom use. Each data source is described below.

### Crowdsourced Online Convenience Sample

Using Amazon Mechanical Turk (mTurk), a crowdsourcing platform for data collection (Chandler and Shapiro, 2016; Miller et al., 2017; Mortensen and Hughes, 2018; Peer et al., 2014; Strickland and Stoops, 2018; 2019), we notified people with registered mTurk accounts between September 2020 and March 2021 that they could complete a screening questionnaire to determine their eligibility for a large online survey pertaining to drug use and social conditions. People were eligible for inclusion into that survey study convenience sample if they were >18 years, US residents, English language proficient, reported using: alcohol only (nicotine and caffeine use permitted), opioids (licit or illicit), and illicit stimulants during the 6 month period prior to screening (people reporting opioid and/stimulant use could report other drug use and remain eligible). Because the survey did not solicit personally identifiable information, the study was considered exempt by the National Institutes of Health Institutional Review Board (NIH IRB). For a more detailed description of the methods, see Smith et al., 2021c; Smith et al., 2021d.

### Convenience Sample Survey Measures

Items assessed included basic demographic information (age, gender, race/ethnicity, highest education attained, employment status, annual income and zip code), lifetime and past-year substance use, DSM-5 SUD symptom checklist for all diagnostic items, and a single-item question asking respondents to indicate whether they had ever received SUD treatment. Lifetime nonmedical use of opioids was defined as any medically unsupervised use of prescription opioids, heroin, or fentanyl. For modeling purposes and to increase concordance with measures employed by NSDUH, age was coded as under versus over 35. Sex/gender was coded as male versus nonmale (an arbitrary, admittedly imperfect solution to the small cell size for respondents who identified as nonbinary).

To test for greater kratom use likelihood among people who could reasonably be described as “young, white, and at least middle class,” we created an indicator variable for both men and women who were: under the age of 35 years, of White race/ethnicity, at least high school educated, employed, and making above US poverty line annual household incomes.

Rural and metropolitan classifications were assigned according to the 2013 rural-urban continuum (Beale) codes, a classification scheme primarily developed by the United States Department of Agriculture (USDA) that classifies counties as being “large metropolitan”, “small metropolitan”, or “non-metropolitan” and the degree to which each is influenced by population size, metropolitan area, urbanization, or adjacency to a metro area. For our online convenience sample, we converted participants’ zip codes to county-level codes and assigned respondents to one of the three aforementioned categories, reflecting the county they reported residing in for the majority of the past year.

### Nationally Representative Sample

Data from the 2019 NSDUH (questionnaire items on kratom use were included in the NSDUH for the first time in 2019) included survey responses from a nationally representative US sample of persons aged 12 and older. Here, we included only responses from persons >18 years of age. The NSDUH employs a “probability proportional to size” sampling design to collect responses from noninstitutionalized civilians in all 50 states and the District of Columbia, but not from people who are housing insecure, incarcerated, institutionalized, or actively deployed in military service. These data are therefore considered representative for approximately 97% of the US population (Lofquist et al., 2012). Analysis of publicly available NSDUH data is also considered exempt from institutional review by the NIH IRB.

### NSDUH Survey Items

We used all measures from the NSDUH that were concordant with measures from our online convenience sample: basic demographic information (age, sex/gender, race/ethnicity, highest education attained, employment status, annual income), an indicator variable for lifetime nonmedical use of opioids (either prescription opioids or heroin), ever having received SUD treatment, and an indicator variable for past-year SUD (by DSM-IV criteria: see next paragraph). NSDUH provides a recoded variable of rural-urban continuum codes (COUTYP4) in which people are classified as living in a large metro, small metro, or non-metro country in the same fashion as in our online convenience sample. We constructed a “young, white, and at least middle class” indicator variable in the same fashion as in our convenience sample.

To ensure maximal comparability of the analyses from the two data sources, we dichotomized demographic variables to match exactly, and we selected concordant indicators of substance use. The only included variable that differed between data sources was the indicator for moderate to severe past-year SUD. In our online convenience sample, past-year SUD was measured using a DSM-5 checklist for SUD for any substance by endorsing >3 DSM-5 SUD diagnostic criteria. Participants were prompted to complete the DSM-5 SUD checklist for one of two conditions: 1) for the substance (alcohol included) they believed they had the biggest problem with during the past year or 2) for those who did not believe they had any alcohol/drug problems, for the substance they had used most frequently. Those endorsing >3 diagnostic criteria were coded moderate-severe). Because NSDUH does not administer the DSM-5 SUD questionnaire, we selected a proxy variable (UDPYILL) that indicates past year DSM-IV dependence on or abuse of an illicit substance.

### Analytic Plan

We generated descriptive results, displayed in Table 1, for the full online convenience sample and the subsets of participants reporting lifetime and past year kratom use. For the 2019 NSDUH data, we describe the sample by reporting nationally representative proportion estimates of lifetime and past year kratom use split by demographic factors and substance use factors associated with kratom use in Table 2.

**TABLE 1:** Demographics for our online crowdsourced sample, by lifetime and past-year kratom use.

	Complete sample		Lifetime kratom use		Past-year kratom use	
	N	M ± SD	N	M ± SD	N	M ± SD
Age	2,615	36.65 ± 11.35	289	33.58 ± 8.67	174	33.58 ± 8.67
	*N*	*%*	*N*	*%*	*N*	*%*
Young Age (<35 Years)	1,335	51.05	179	61.94	103	59.20
Sex/gender						
Male	1,052	40.23	154	53.29	87	50.00
Female	1,531	58.55	126	43.60	81	46.55
Nonbinary	32	1.22	9	3.11	6	3.45
Race/Ethnicity						
White	1954	74.72	209	72.32	124	71.26
US Minority	661	25.28	80	27.68	50	28.74
Education						
HS Graduate	1,199	45.85	189	65.40	115	66.09
College Graduate	1,416	54.15	100	34.60	57	32.76
Employment						
Employed	2054	78.55	216	74.74	125	71.84
Unemployed	561	21.45	73	25.26	49	28.16
Annual Income						
Below Poverty Line	541	20.69	79	27.34	47	27.01
Above Poverty Line	2074	79.31	210	72.66	127	72.99
Rural-Urban Continuum						
Large Metro	1,398	53.46	137	47.40	81	46.55
Small Metro	797	30.48	93	32.18	55	31.61
Non-Metro	420	16.06	59	20.42	38	21.84
Ever SUD Treatment	284	10.86	82	28.37	46	26.44
Moderate - Severe SUD	949	36.29	193	66.78	117	67.24
Lifetime NMO	801	30.63	209	72.32	129	74.14
“White Middle-Class” indicator						
Male	289	11.05	48	16.61	25	14.37
Nonmale (Female or nonbinary)^a^	341	13.04	31	10.73	18	10.34

a Our use of “male” as the reference category, with female and nonbinary collapsed into the other category, was our admittedly imperfect solution to the smallness of the cell size for respondents identifying as nonbinary in our survey. Despite misgivings about the categorization, we think it is preferable to excluding respondents who did not fall into one of the two large categories. The issue did not arise for the nationally representative NSDUH, data **(Tables 2, 5, 6)** because the NSDUH, survey did not included “nonbinary” as a response choice.

**TABLE 2:** Survey-weighted proportions of respondents with lifetime and past-year kratom use, National Survey on Drug Use and Health (NSDUH), 2019.

	Past-year kratom use		Lifetime kratom use	
	Proportion	95% CI	Proportion	95% CI
Sex/gender				
Female	0.006	[0.005, 0.007]	0.011	[0.010, 0.012]
Male	0.009	[0.008, 0.011]	0.019	[0.016, 0.021]
Race/Ethnicity				
Minority	0.004	[0.003, 0.004]	0.007	[0.006, 0.009]
White	0.009	[0.008, 0.011]	0.019	[0.018, 0.021]
Age				
Under 35	0.011	[0.009, 0.012]	0.022	[0.020, 0.012]
Over 35	0.005	[0.004, 0.006]	0.010	[0.009, 0.021]
Education				
Neither	0.005	[0.003, 0.008]	0.010	[0.007, 0.014]
High School Educated	0.007	[0.005, 0.008]	0.013	[0.011, 0.016]
College Educated	0.008	[0.007, 0.009]	0.017	[0.015, 0.019]
Employment				
Unemployed	0.005	[0.004, 0.006]	0.011	[0.010, 0.013]
Employed	0.009	[0.008, 0.010]	0.017	[0.016, 0.019]
Annual Income				
Below Poverty Line	0.007	[0.005, 0.009]	0.016	[0.013, 0.020]
Above Poverty Line	0.007	[0.006, 0.008]	0.014	[0.013, 0.016]
Rural-urban Continuum				
Rural Zip Code	0.008	[0.005, 0.013]	0.015	[0.011, 0.020]
Urban Zip Code	0.007	[0.006, 0.008]	0.015	[0.013, 0.016]
Lifetime Non-Med Opioid Use				
Yes	0.034	[0.028, 0.040]	0.078	[0.070, 0.086]
No	0.004	[0.004, 0.005]	0.008	[0.007, 0.009]
Past-year Drug Dependence/Abuse				
Yes	0.060	[0.046, 0.078]	0.134	[0.108, 0.165]
No	0.006	[0.005, 0.007]	0.012	[0.011, 0.013]
Lifetime SUD Treatment				
Yes	0.031	[0.024, 0.039]	0.068	[0.057, 0.081]
No	0.006	[0.005, 0.007]	0.011	[0.010, 0.012]
“White Middle-Class” indicator				
Male	0.020	[0.013, 0.029]	0.049	[0.040, 0.061]
Nonmale	0.014	[0.010, 0.019]	0.024	[0.017, 0.032]

**FIGURE 1:**
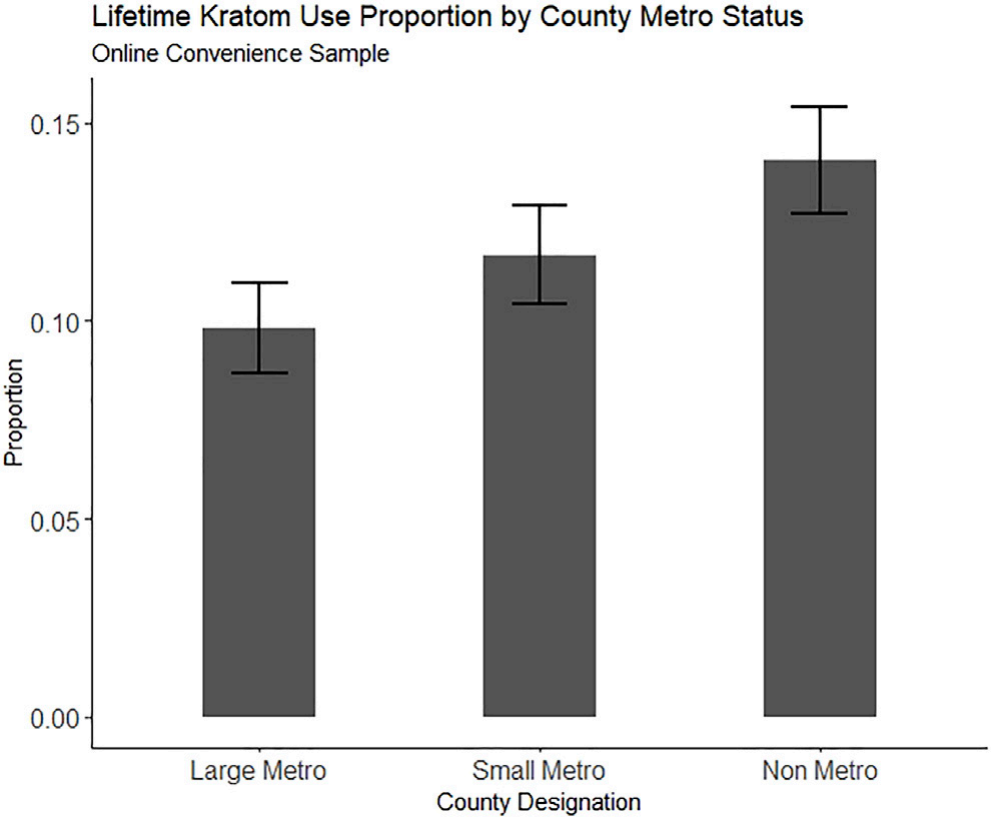
Proportion of people reporting lifetime kratom use in an online convenience sample (N = 2,615), split by county metro status. Error bars represent the estimate 95% confidence interval.

Because one primary aim was to examine regionality, we fit multinomial logistic regression models predicting both lifetime and past-year kratom use from the metropolitan classification of participants’ residence while controlling for demographic factors and substance use factors that have been previously associated with kratom use. Survey sampling weights and survey design-based variance estimation were employed on all NSDUH models to produce nationally representative estimates. All analyses were conducted using R version 4.1.1. The R analyses syntax and datasets generated and analyzed from the NSDUH 2019 dataset for this study can be found on Open Science Framework at doi: 10.17605/OSF.IO/M7DW4 [https://osf.io/m7dw4/]. Lifetime and past-year kratom use proportion estimates for the online convenience sample and NSDUH 2019 are plotted in Figures 1 through 4, respectively.

**FIGURE 2:**
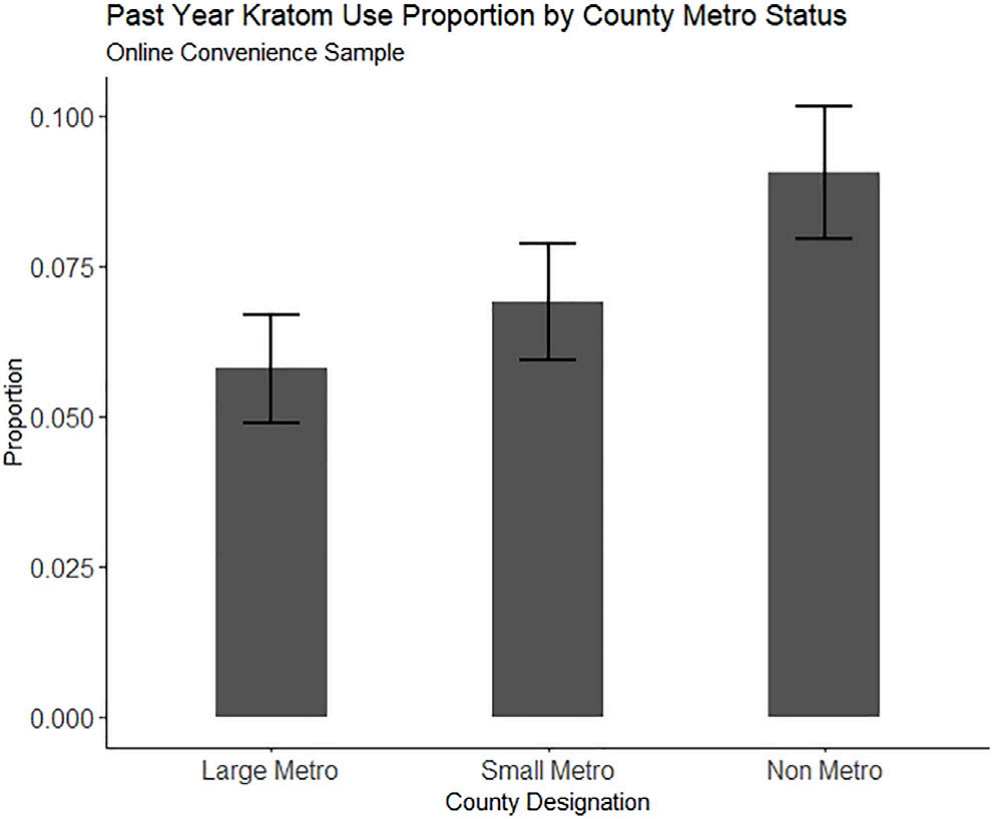
Proportion of people reporting past-year kratom use in an online convenience sample (N = 2,615), split by county metro status. Error bars represent the estimate 95% confidence interval.

**FIGURE 3:**
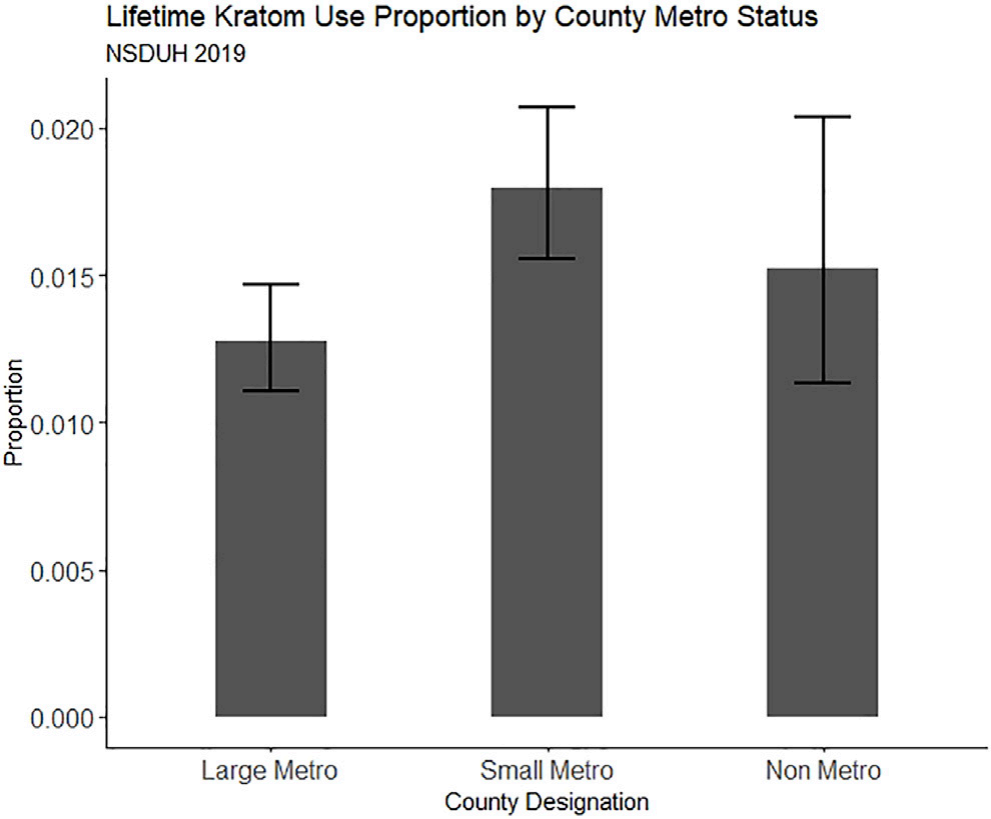
Proportion of people reporting lifetime kratom use in NSDUH 2019, split by county metro status. Error bars represent the estimate 95% confidence interval.

**FIGURE 4:**
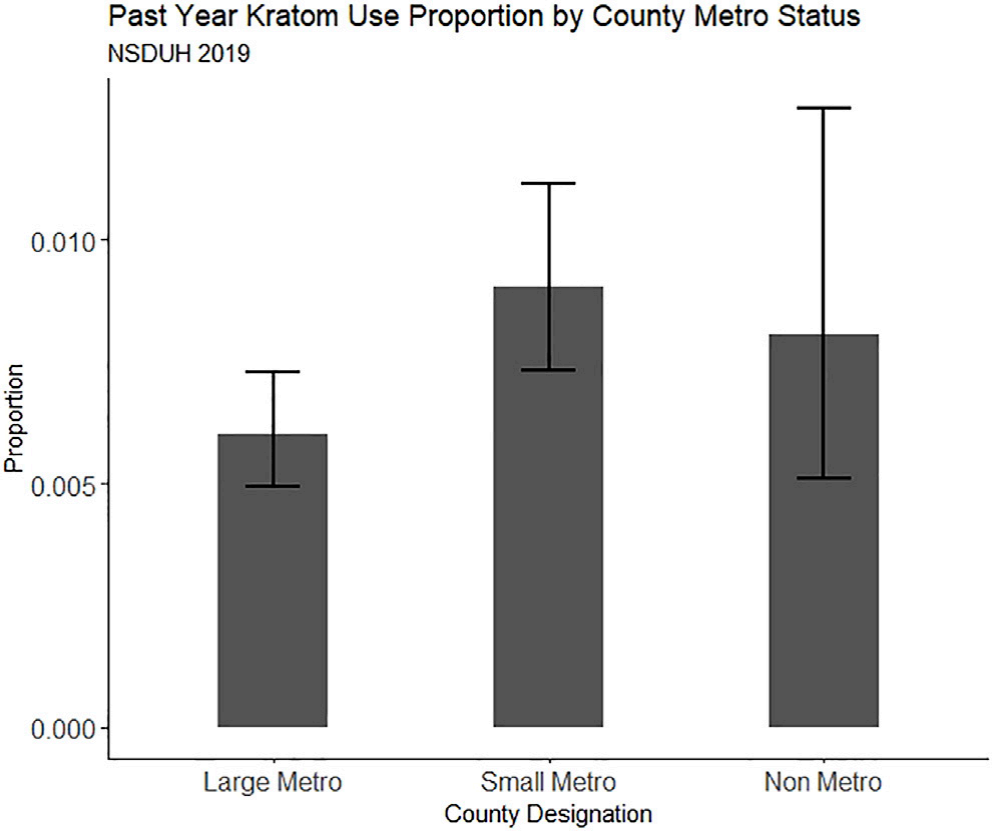
Proportion of people reporting past-year kratom use in NSDUH 2019, split by county metro status. Error bars represent the estimate 95% confidence interval.

Additionally, because we were interested in detecting a signal for higher kratom use prevalence among white, middle-class men and women, we fit two multiple-logistic-regression models from each data set predicting lifetime and past year kratom use from a single combined factor indicating the aforementioned population while controlling for the same non-demographic terms entered into the previously employed models.

All logistic regression model results are displayed in Table 3 through Table 6. Regression coefficients are Table 4 reported as odds ratios and their 95% confidence intervals. We computed variance inflation factors for each predictor term, also displayed in the tables, to ensure that multicollinearity did not substantially influence predictor performance.

**TABLE 3:** Multiple logistic regression models using online crowdsourced data to examine the relationship between county residence and kratom use while controlling for demographic and substance use factors.

Lifetime kratom use - mTurk	OR	95% CI		Z	p	VIF
		Lower	Upper			
Intercept	0.02	0.00	0.03	-17.77	<0.001	
Young Age (<35)	**1.64**	1.24	2.16	3.52	**<0.001**	1.03
Sex/gender (Male – Nonmale)	**1.79**	1.37	2.34	4.24	**<0.001**	1.02
Race (White - US Minority)	0.81	0.60	1.09	-1.39	0.16	1.01
Education (Highschool - College)	**1.39**	1.04	1.87	2.20	**0.03**	1.16
Employed (Unemployed - Employed)	1.10	0.79	1.54	0.55	0.58	1.16
Below Poverty Line Annual Income	1.01	0.72	1.40	0.03	0.98	1.20
Rural - Urban Continuum						
Non-metro - Large Metro	1.23	0.95	1.59	1.57	0.12	1.05
Small Metro - Large Metro	1.04	0.82	1.32	0.32	0.75	1.05
Lifetime Non-Medical Opioid Use	**5.13**	3.80	6.94	10.62	**<0.001**	1.17
Moderate to Severe SUD	**2.00**	1.49	2.68	4.61	**<0.001**	1.17
Lifetime SUD Treatment	**1.53**	1.09	2.14	2.47	**0.01**	1.16

Past-Year Kratom Use - mTurk	OR	95% CI		Z	p	VIF
		Lower	Upper			
Intercept	0.02	0.00	0.03	-15.97	<0.001	
Young Age (<35)	1.37	0.98	1.90	1.84	0.07	1.03
Sex/gender (Male - Nonmale)	**1.47**	1.06	2.03	2.29	**0.02**	1.03
Race (White - US Minority)	0.77	0.54	1.10	-1.42	0.15	1.01
Education (Highschool - College)	**1.44**	1.00	2.08	1.97	**0.05**	1.17
Employed (Unemployed - Employed)	1.30	0.88	1.93	1.31	0.19	1.17
Below Poverty Line Annual Income	0.92	0.61	1.37	-0.42	0.67	1.20
Rural - Urban Continuum						
Non-metro - Large Metro	1.26	0.93	1.72	1.51	0.13	1.05
Small Metro - Large Metro	1.08	0.80	1.44	0.49	0.62	1.05
Lifetime Non-Medical Opioid Use	**5.22**	3.56	7.66	8.46	**<0.001**	1.17
Moderate to Severe SUD	**1.94**	1.34	2.79	3.54	**<0.001**	1.18
Lifetime SUD Treatment	1.20	0.80	1.79	0.87	0.39	1.16

*χ* ^*2*^(11) = 331.38; Pseudo-R^2^, 0.24; *p* = <0.01; AIC, 1,510.53. *χ* ^*2*^(11) = 192.74; Pseudo-R^2^, 0.18; *p* = <0.01; AIC, 1,110.48. Statistically significant explanatory variables are denoted by bolded text.

**TABLE 4:** Multiple logistic regression models using online crowdsourced data to examine the relationship between a “white middle-class” indicator and kratom use while controlling for substance use factors.

Lifetime kratom use - mTurk	OR	95% CI		Z	p	VIF
		Lower	Upper			
Intercept	0.03	0.03	0.04	-24.09	<0.001	
White Middle-Class indicator						
Male	**1.94**	1.33	2.82	3.45	**<0.001**	1.03
Nonmale	0.89	0.58	1.36	-0.54	0.59	1.03
Rural - Urban Continuum						
Non-metro - Large Metro	1.25	0.97	1.60	1.72	0.09	1.02
Small Metro - Large Metro	1.05	0.83	1.33	0.40	0.69	1.02
Lifetime Non-Medical Opioid Use	**5.41**	4.02	7.28	11.16	**<0.001**	1.14
Moderate to Severe SUD	**2.22**	1.66	2.96	5.41	**<0.001**	1.14
Lifetime SUD Treatment	**1.56**	1.12	2.18	2.66	**0.01**	1.14

Past-year Kratom Use - mTurk	OR	95% CI		Z	p	VIF
		Lower	Upper			
Intercept	0.02	0.01	0.03	-21.77	<0.001	
White Middle-Class indicator						
Male	1.45	0.90	2.32	1.54	0.12	1.02
Nonmale	0.83	0.49	1.40	-0.71	0.48	1.02
Rural - Urban Continuum						
Non-metro - Large Metro	1.30	0.96	1.75	1.70	0.09	1.02
Small Metro - Large Metro	1.09	0.81	1.45	0.56	0.58	1.02
Lifetime Non-Medical Opioid Use	**5.56**	3.81	8.11	8.91	**<0.001**	1.15
Moderate to Severe SUD	**2.13**	1.49	3.05	4.12	**<0.001**	1.15
Lifetime SUD Treatment	1.24	0.83	1.84	1.06	0.29	1.13

*χ* ^*2*^(7) = 179.39; Pseudo-*R* ^2^, 0.17; *p* = <0.01; *AIC*, 1,115.83. *χ* ^*2*^(7) = 304.80; Pseudo-*R* ^2^, 0.22; *p* = <0.01; *AIC*, 1,529.11. Statistically significant explanatory variables are denoted by bolded text.

## Results

### Crowdsourced Online Convenience Sample

Between September 2020 and March 2021 a total of 13,608 people completed screening questionnaires on mTurk, 3,414 (25.1%) meet study inclusion criteria and were invited to participate, 2,864 (21.0%) completed the survey, and 2,615 (19.2%) passed all data quality checks and constituted the final analyzable sample.

Table 1 shows descriptive data. The respondents were majority female (58.6%), White (74.7%), college educated (54.2%), employed (78.6%), and living in large metropolitan counties (53.5%). Lifetime kratom use was reported by 289 (11.1%) respondents. Past-year kratom use was reported by 174 (6.7%). The subset of participants who reported lifetime and past-year kratom use contained a higher proportion of male, high school-educated people, people making below US poverty line annual incomes, people living in non-metro (rural) counties, people having ever received SUD treatment, and people meeting criteria for a severe SUD.

Increased likelihood of lifetime kratom use was predicated by young (<35) age (OR = 1.64, 95% CI = 1.24, 2.16), male sex/gender (OR = 1.79, 95% CI = 1.37, 2.34), being high school educated (OR = 1.39, 95% CI = 1.04, 1.87), lifetime nonmedical use of opioids (OR = 5.13, 95% CI = 3.80, 6.94), at least moderate SUD (OR = 2.00, 95% CI = 1.49, 2.68), and having ever received SUD treatment (OR = 1.53, 95% CI = 1.09, 2.14).

Similarly, increased likelihood of past-year kratom use was indicated by male sex/gender (OR = 1.47, 95% CI = 1.06, 2.03), being high school educated (OR = 1.44, 95% CI = 1.00, 2.08), lifetime nonmedical use of opioids (OR = 5.22, 95% CI = 3.56, 7.66), and at least moderate SUD (OR = 1.94, 95% CI = 1.34, 2.79).

Residence in a rural county was not significantly associated with either lifetime or past-year kratom use in our crowdsourced sample, although there was a trend towards an association of non-metro county residence with increased odds of lifetime (OR = 1.25, 95% CI = 0.97, 1.60) and past-year kratom use (OR = 1.30, 95% CI = 0.96, 1.75).

The “White, middle-class” indicator was associated with increased likelihood of lifetime kratom use among men (OR = 1.94, 95% CI = 1.33, 2.82) but not women or nonbinary respondents (OR = 0.89, 95% CI = 0.58, 1.36). Past-year kratom use was not associated with the “White, middle-class” indicator for either sex/gender category.

### Nationally Representative Sample

Table 2 shows weighted prevalence estimates for lifetime and past-year kratom use nested within demographic and substance use factors. For complete model results, see Table 5 and Table 6.

**TABLE 5:** Survey-weighted multiple logistic regression models using nationally representative NSDUH 2019 data to examine the relationship between county residence and kratom use while controlling for demographic and substance use factors.

Lifetime kratom use - NSDUH	OR	95% CI		t	p	VIF
		Lower	Upper			
Intercept	0.00	0.00	0.00	-25.17	<0.001	
Young age (< 35 years)	**2.25**	1.79	2.83	6.93	**<0.001**	1.33
Sex/gender (Male - Female)	**1.41**	1.14	1.73	3.24	**<0.001**	1.44
Race/ethnicity (White - US Minority)	**2.41**	1.91	3.03	7.49	**<0.001**	2.07
Education						
Highschool Grad - Not Highschool Grad	1.20	0.77	1.86	0.80	0.43	1.65
College Grad - Not Highschool Grad	1.55	1.00	2.40	1.94	0.06	1.65
Employment (Employed - Unemployed)	**1.22**	1.01	1.48	2.08	**0.04**	1.24
Below Poverty Line Annual Income	1.19	0.93	1.52	1.41	0.17	1.57
Rural - Urban Continuum						
Non-metro - Large Metro	1.11	0.79	1.56	0.59	0.56	2.41
Small Metro - Large Metro	**1.32**	1.05	1.66	2.33	**0.03**	2.41
Lifetime Non-Medical Opioid Use	**6.32**	5.19	7.69	18.40	**<0.001**	1.50
Past Year Drug Dependence/Abuse	**3.17**	2.33	4.33	7.31	**<0.001**	1.46
Lifetime SUD Treatment	**2.07**	1.57	2.71	5.21	**<0.001**	1.96

Past-year Kratom Use - NSDUH	OR	95% CI		t	p	VIF
		Lower	Upper			
Intercept	0.00	0.00	0.00	-21.19	0.00	
Young age (< 35 years)	**2.06**	1.59	2.68	5.40	**<0.001**	1.92
Sex/gender (Male - Female)	1.29	0.95	1.76	1.64	0.11	1.80
Race/ethnicity (White - US Minority)	**2.21**	1.69	2.88	5.85	**<0.001**	1.28
Education						
Highschool Grad - Not Highschool Grad	1.15	0.66	2.02	0.50	0.62	3.36
College Grad - Not Highschool Grad	1.35	0.79	2.28	1.10	0.28	3.36
Employment (Employed - Unemployed)	**1.52**	1.14	2.02	2.88	**0.01**	2.06
Below Poverty Line Annual Income	1.01	0.69	1.49	0.06	0.95	2.04
Rural - Urban Continuum						
Non-metro - Large Metro	1.26	0.74	2.16	0.86	0.40	3.95
Small Metro - Large Metro	**1.41**	1.03	1.93	2.17	**0.04**	3.95
Lifetime Non-Medical Opioid Use	**4.62**	3.36	6.37	9.40	**<0.001**	2.01
Past Year Drug Dependence/Abuse	**2.99**	2.11	4.24	6.17	**<0.001**	1.57
Lifetime SUD Treatment	**2.02**	1.40	2.90	3.77	**<0.001**	2.16

Pseudo *R* ^*2*^ = 0.12; *p* = <0.01; AIC = 3,922.74; Est. Dispersion Parameter = 0.99. Pseudo *R* ^*2*^ = 0.17; *p* = <0.01; AIC = 6,744.25; Est. Dispersion Parameter = 0.98. Statistically significant explanatory variables are denoted by bolded text.

**TABLE 6:** Survey-weighted multiple logistic regression models using nationally representative NSDUH 2019 to examine the relationship between a “white middle-class” indicator and kratom use while controlling for substance use factors.

Lifetime kratom use - NSDUH	OR	95% CI		t	p	VIF
		Lower	Upper			
Intercept	0.01	0.00	0.01	-55.52	<0.001	
White Middle-Class indicator						
Male	**3.10**	2.44	3.93	9.31	**<0.001**	1.11
Female	**1.86**	1.32	2.60	3.57	**<0.001**	1.37
Rural - Urban Continuum						
Non-metro - Large Metro	1.22	0.89	1.69	1.22	0.23	1.24
Small Metro - Large Metro	**1.41**	1.12	1.77	2.94	**<0.001**	1.24
Lifetime Non-Medical Opioid Use	**6.77**	5.53	8.29	18.56	**<0.001**	1.45
Past Year Drug Dependence/Abuse	**3.50**	2.56	4.79	7.83	**<0.001**	1.60
Lifetime SUD Treatment	**2.11**	1.61	2.75	5.48	**<0.001**	1.64

Past-year Kratom Use - NSDUH	OR	95% CI		t	p	VIF
		Lower	Upper			
Intercept	0.00	0.00	0.00	−49.11	<0.001	
White Middle-Class indicator						
Male	**2.30**	1.59	3.33	4.41	**<0.001**	1.05
Female	**2.05**	1.51	2.79	4.61	**<0.001**	1.10
Rural - Urban Continuum						
Non-metro - Large Metro	1.36	0.82	2.28	1.19	0.24	1.37
Small Metro - Large Metro	**1.49**	1.09	2.04	2.48	**0.02**	1.37
Lifetime Non-Medical Opioid Use	**5.03**	3.66	6.93	9.91	**<0.001**	1.61
Past Year Drug Dependence/Abuse	**3.27**	2.30	4.64	6.63	**<0.001**	1.37
Lifetime SUD Treatment	**2.01**	1.39	2.90	3.73	**<0.001**	1.54

Pseudo-*R* ^*2*^ = 0.16; *p* = <0.01; AIC = 6,945.21; Est. Dispersion Parameter = 0.95. Pseudo-*R* ^*2*^ = 0.16; *p* = <0.01; AIC = 6,945.21; Est. Dispersion Parameter = 0.95. Statistically significant explanatory variables are denoted by bolded text.

In these models, which duplicated as closely as possible the models we used for our online crowdsourced sample, increased likelihood of lifetime kratom use was associated with young age (OR = 2.25, 95% CI = 1.79, 2.83), male sex/gender (OR = 1.41, 95% CI = 1.14, 1.73), White race/ethnicity (OR = 2.41, 95% CI = 1.91, 3.03), being employed (OR = 1.22, 95% CI = 1.01, 1.48), lifetime nonmedical use of opioids (OR = 6.34, 95% CI = 5.19, 7.69), past-year drug dependence or abuse (OR = 3.17, 95% CI = 2.33, 4.33), and having ever received SUD treatment (OR = 2.07, 95% CI = 1.57, 2.71).

Findings for past-year kratom use differed slightly from those for lifetime use; past-year use was associated with young age (OR = 2.06, 95% CI = 1.59, 2.68), White race/ethnicity (OR = 2.21, 95% CI = 1.69, 2.88), being employed (OR = 1.52, 95% CI = 1.14, 2.02), lifetime nonmedical use of opioids (OR = 4.62, 95% CI = 3.36, 6.37), past-year drug dependence/abuse (OR = 2.99, 95% CI = 2.11, 4.24), and having ever received SUD treatment (OR = 2.02, 95% CI = 1.40, 2.90).

Compared to those residing in large metro counties, those in small metro counties had greater odds of both lifetime (OR = 1.32, 95% CI = 1.05, 1.66) and past-year (OR = 1.41, 95% CI = 1.03, 1.93) kratom use in NSDUH 2019 binomial models. Also, unlike in the convenience sample, we found greater odds of lifetime (OR = 3.10, 95% CI = 2.44, 3.93) and past-year kratom use (OR = 2.30, 95% CI = 1.59, 3.33) among men categorized as “White and at least middle class.” For women categorized as “White and at least middle class,” the association was similar but weaker, for both lifetime (OR = 1.86, 95% CI = 1.32, 2.60) and past-year (OR = 2.05, 95% CI = 1.51, 2.79) kratom use.

## Discussion

We aimed to determine whether rurality was associated with lifetime or past-year kratom use while controlling for potentially confounding factors, and, at the same time, whether there was an emerging subpopulation of kratom users who we believe are increasingly being exposed to kratom-related media content, namely younger, White, educated, employed people. The use of two separate data sources helps increase confidence in our findings.

### Rurality, Opioid-Related Harms, and Kratom Use

Though there is substantial survey evidence indicating that kratom is often used as a form of self-managed MOUD (Grundmann, 2017; Smith and Lawson, 2017; Coe et al., 2019; Garcia-Romeu et al., 2020)—for which the need might be greatest in rural communities—we did not find a significant association between past-year rural residence and kratom use. We did, however, find that residency in a small metro (suburban) county was associated with a 32% greater likelihood of lifetime kratom use and a 41% greater likelihood of past-year kratom use. Our ability to detect an association between rurality and kratom use may have been hindered by rural/urban classification in the NSDUH data and the relatively small proportion of rural-residing respondents in our online convenience sample. The NSDUH dataset provides only three levels of rural-urban classification: large metro, small metro, and non-metro. We can only conclude that non-metro residents do not display increased odds of kratom use compared with those living in metro counties, and cannot attest to varying degrees of rurality in comparison to the varying degrees of metropolitan size and their association (or lack thereof) with kratom use. Previous investigations suggest that opioid-related harms are relatively greater, and subsequent public health policy response is relatively slower, in the most remote US counties (Thomas et al., 2020; Andrilla and Patterson, 2021). Thus, it is important for future work in this realm to distinguish between the most rural counties and those closer to metropolitan areas. Here, we approached an approximation in measurement, but more granular work will be needed as kratom products continue to be uniquely branded, marketed, and sold in the US.

### Kratom Use and the White Middle Class

In our online convenience sample, we observed significantly greater odds of lifetime kratom use among White men, and greater odds of past-year kratom use among men only. When examining combined factor(s) through which we operationalized “White and middle-class,” we found that men in the White, middle-class group were nearly twice as likely to report lifetime kratom use as other men. Further, we observed much stronger and consistent associations between this indicator and both lifetime and past-year kratom use in NSDUH data. Lifetime kratom use was 3.10 times more likely to be reported by White, middle-class men and 1.86 times as likely to be reported by White, middle-class women. With respect to past-year kratom use, White, middle-class men and women were 2.30 times and 2.05 times as likely to report use, respectively. We suspect that these kratom users are not only of people with SUD histories, but also people who represent far more socially normative substance-use sub-groups who are using kratom for wellness purposes or enhancement (e.g., to boost cognitive and physical performance), as these motivations have been expressed by kratom-using people in prior investigations (Smith et al., 2021a; Smith et al., 2021b). Given the cross-sectional nature of these data and that these analyses are the first to use a “White, middle-class” indicator variable to represent a specific demographic of kratom users, we cannot claim that kratom use is increasing among this demographic. Rather, we can only assert that kratom use prevalence is significantly higher with this demographic intersection, seemingly among those with suburban residence, when compared with the rest of the US population (using NSDUH data), or when compared to other survey respondents with normative and illicit substance use (in our crowdsourced convenience sample). That kratom use has been associated with similar “middle class” attributes in kratom-specific online surveys in the US suggests that at least a sizeable proportion of people using kratom can be characterized in this way, even though we do not dismiss the heterogeneity that likely exists within this group. For instance, there are people who use kratom to address anxiety, chronic pain, fatigue, or SUD who are also among such a demographic group, but this does not suggest that there are no other motivations within this demographic groups, or other demographic groups for whom kratom use will become more prevalent for these or other reasons. With respect to the former, the lack of greater understanding of heterogeneity of kratom-using people may be an artifact of the questions that have been typically asked in surveys.

### Does it all Just Come Back to Opioids?

The kratom narrative in the US is increasingly framed as part of a broader opioid narrative. Here, while we see greater rates of lifetime and past-year kratom use among people we have conceptualized as a “White, suburban middle class,” these analyses still provide strong support for the association between kratom use and nonmedical use of prescribed or illicit opioids. Both our convenience sample models and our nationally representative models indicate that people who have ever used opioids nonmedically display five times greater likelihood of having used kratom in the past year. We also saw a smaller but still sizeable set of associations between past-year kratom use and moderate to severe SUD, past-year drug dependence/abuse, and having ever received SUD treatment.

Again, the cross-sectional nature of our data and that of others who have found similar associations (Grundmann et al., 2021; Palamar, 2021; Schimmel et al., 2021) prevent us from speculating as to which preceded the other. Currently, kratom use has yet to predict incident SUD at later time points (except for the logical inevitability of its having to precede kratom use disorder, a diagnostic entity that is not yet formally recognized but has been documented by our group and others). Because kratom is often used by people to mitigate or reduce symptoms of OUD or other SUDs, including withdrawal, we know that at least some portion of people initiating kratom use are doing so only after initiating nonmedical use of opioids.

### Importance of Sampling to Current and Future Kratom Research

As noted above, though we have found some evidence of greater kratom use among people that we operationalize as being “White and middle class” and who reside in small metro (“suburban”) areas in the US, kratom-using people remain a heterogenous group in terms of motivation(s) for use (which may be shifting and which are likely dynamic) and substance use history/experience. The differences we observed in comparing results from our two data sources highlight the importance of improving how we study kratom use and the people who use it. This includes purposeful sampling, improved survey methods (and survey question wording), investing in longitudinal study designs, and adopting real-time ambulatory assessment where possible. All of these can help to produce a more complete picture than currently exists. Moreover, there is a need for ongoing assessment of the kratom market and changes in the US commercial kratom industry (which we believe will increase and become more diverse in terms of what consumer groups are targeted with unique kratom product branding). In our online convenience sample, which contained a much greater proportion of people using stimulant and/or opioid drugs than in the US population, we saw that substance use factors (opioid use, SUD, treatment) were the most important variable for explaining the incidence of kratom use. It may be that for such groups, kratom will continue to be marketed in terms of its potential for harm reduction as a form self-managed MOUD. Likewise, when analyzing data representative of the greater US population, we observed greater associations with demographic factors (i.e., the “White middle class” factor) that, when examined in the convenience sample models, did not contribute significantly. It may be that for this group, kratom will come to be marketed as a wellness or energy-enhancing supplement that is specific to boosting performance (e.g., pre-workout, “nootropic”). These and other subgroups are likely to be identified as research continues. In the interim, continuing methods such as the ones we used here, wherein we analyzed data from two unique survey sources, have clear benefits: our convenience sample provided us with greater insight into the nuances that may exist among people who use illicit substances, whereas the NSDUH sample provided greater insight into substance use phenomena among the US population as a whole. Ultimately both converged to suggest that kratom use is, for now, a mostly middle-class and suburban phenomenon with possibly greater prevalence among men. However, given kratom’s relative novelty in the US, this is subject to change, making continued assessment critical.

## Limitations

Findings from the analyses here should be interpreted with several limitations in mind, including the cross-sectional nature of the data collection for both our crowdsourced convenience sample using mTurk and the NSDUH survey. While NSDUH 2019 data are considered representative for approximately 97% of US residents, the crowdsourced convenience sample contains greater proportions of White people and people earning more than $50,000 USD than the US population, which could hinder the results’ generalizability to people from US minority communities. Each may limit generalizability to the larger kratom-using community in the US: mTurk represents only one platform for crowdsourcing (and our sampling strategy included only people who reported past-6 months alcohol or past 6 months opioid and/or psychostimulant use), and the NSDUH may not have captured respondents who had used kratom, given the survey item wording (which does not reflect all kratom slang names nor all kratom product types), nor as noted earlier, people who were institutionalized, homeless, or in military service.

Our use of similar data collected from two separate sources is, in some respects, a limitation, in that survey item order and, most importantly, phrasing was different. This means that while we examined variables that were similar, they were not measured or collected using identical methods. We believe that the items selected and/or re-coded, including the composite variable created for the “White middle class” factor, were nonetheless meaningfully similar indicators (or were merely identical indicators for such things as age cut-offs). It may be that differences in methodology between the two surveys are more a strength than a limitation, helping to address what has been called a “generalizability crisis” in behavioral research (Yarkoni, 2020).

Perhaps the greatest limitation of the findings presented here is also among the greatest limitations to all self-report kratom survey research: we ultimately cannot be sure what respondents meant when they reported having used “kratom,” as this term could have meant many different things practically and experientially: alkaloid content varying among specific types of kratom products (e.g., different leaves were used to make different batches of a product at different times by different distributors or vendors), variation between kratom products (e.g., extracts, loose leaf, pulverized plant matter), and different route of administration or dosing (e.g., slowly sipping tea versus consuming one kratom shot versus ingesting prepared capsules). A related limitation is that motivations for kratom use among respondents were not measured, leaving us only to speculate based on prior literature and changes in kratom product marketing.

## Future Directions

All nationally representative surveys designed to assess substance use prevalence should now assess for kratom use. Unlike synthetic novel psychoactive substances, kratom remains largely legal in the US and has not resulted in widespread reports of misuse or toxicity, relative to novel synthetics or even traditional illicit drugs (e.g., heroin, cocaine). This means that there remains the potential for kratom to become adopted into US culture with less stigma than other emerging psychoactive substances, possibly facilitating the perception of kratom to be more like cannabis, alcohol, or caffeine—substances that may produce psychological or physical dependence, but which are perceived by some users to be mostly compatible with or even helpful to improving the quality of everyday life (e.g., work, recreation; Smith et al., 2021b; Smith et al., 2021d). Although we cannot be certain, there are no clear indicators that kratom use is decreasing in the US. Rather, it seems that kratom products are poised to be used by a more diverse group, in that kratom is now being framed not only a self-treatment for psychiatric or SUD symptoms, but as a means for enhancing mood, performance, and recreation. Given the unknown current and future prevalence of kratom use, and the limitations of any single survey method, we recommend that future survey data, wherever possible, be analyzed with data collected by similar, but still distinct, methods in order to increase the ability to detect similar (or dissimilar) findings of public health significance. While we cannot claim that any finding from one of the datasets used here validated another in the strictest sense, we do believe that when methods such as these are repeated often enough there will be clear signals that are apparent and which can be followed up on using more precise methods, which are desperately needed in this nascent body of research.

## Data Availability

The R syntax and datasets generated and analyzed from the NSDUH 2019 dataset for this study can be found on Open Science Framework at DOI: 10.17605/OSF.IO/M7DW4 [https://osf.io/m7dw4/].

## References

[B1] Andrilla C. H. A. Coulthard C. Patterson D. G. (2018). Prescribing Practices of Rural Physicians Waivered to Prescribe Buprenorphine. Am. J. Prev. Med. 54 (6), S208–S214. 10.1016/j.amepre.2018.02.006 29779544

[B2] Andrilla C. H. A. Patterson D. G. (2021). Tracking the Geographic Distribution and Growth of Clinicians with a DEA Waiver to Prescribe Buprenorphine to Treat Opioid Use Disorder. J. Rural Health, 1–9. 10.1111/jrh.12569 33733547

[B3] Ayres I. Jalal A. (2018). The Impact of Prescription Drug Monitoring Programs on U.S. Opioid Prescriptions. J. L. Med Ethics 46 (2), 387–403. 10.1177/1073110518782948 30146997

[B4] Babu K. M. McCurdy C. R. Boyer E. W. (2008). Opioid Receptors and Legal Highs: Salvia Divinorum and Kratom. Clin. Toxicol. (Phila) 46 (2), 146–152. 10.1080/15563650701241795 18259963

[B5] Barnett M. L. Lee D. Frank R. G. (2019). In Rural Areas, Buprenorphine Waiver Adoption since 2017 Driven by Nurse Practitioners and Physician Assistants. Health Aff. (Millwood) 38 (12), 2048–2056. 10.1377/hlthaff.2019.00859 31794302 PMC6938159

[B6] Bath R. Bucholz T. Buros A. F. Singh D. Smith K. E. Veltri C. A. (2020). Self-reported Health Diagnoses and Demographic Correlates with Kratom Use: Results from an Online Survey. J. Addict. Med. 14 (3), 244–252. 10.1097/ADM.0000000000000570 31567595 PMC7446542

[B7] Boyer E. W. Babu K. M. Adkins J. E. McCurdy C. R. Halpern J. H. (2008). Self-treatment of Opioid Withdrawal Using Kratom (Mitragynia Speciosa Korth). Addiction 103 (6), 1048–1050. 10.1111/j.1360-0443.2008.02209.x 18482427 PMC3670991

[B8] Boyer E. W. Babu K. M. Macalino G. E. Compton W. (2007). Self-treatment of Opioid Withdrawal with a Dietary Supplement, Kratom. Am. J. Addict. 16 (5), 352–356. 10.1080/10550490701525368 17882605

[B9] Bunting A. M. Oser C. B. Staton M. Eddens K. S. Knudsen H. (2018). Clinician Identified Barriers to Treatment for Individuals in Appalachia with Opioid Use Disorder Following Release from Prison: a Social Ecological Approach. Addict. Sci. Clin. Pract. 13 (1), 23–10. 10.1186/s13722-018-0124-2 30509314 PMC6278109

[B10] Carcache de Blanco E. J. Kinghorn A. D. (2021). “Botanical Dietary Products,” in Remington (Elsevier), 45–58. 10.1016/B978-0-12-820007-0.00003-9

[B11] Chandler J. Shapiro D. (2016). Conducting Clinical Research Using Crowdsourced Convenience Samples. Annu. Rev. Clin. Psychol. 12 (1), 53–81. 10.1146/annurev-clinpsy-021815-093623 26772208

[B12] Coe M. A. Pillitteri J. L. Sembower M. A. Gerlach K. K. Henningfield J. E. (2019). Kratom as a Substitute for Opioids: Results from an Online Survey. Drug Alcohol Depend 202, 24–32. 10.1016/j.drugalcdep.2019.05.005 31284119

[B13] Cole E. S. DiDomenico E. Green S. Heil S. K. R. Hilliard T. Mossburg S. E. (2021). The Who, the what, and the How: A Description of Strategies and Lessons Learned to Expand Access to Medications for Opioid Use Disorder in Rural America. Subst. Abus 42 (2), 123–129. 10.1080/08897077.2021.1891492 33689594

[B14] Fowble K. L. Musah R. A. (2019). A Validated Method for the Quantification of Mitragynine in Sixteen Commercially Available Kratom (Mitragyna Speciosa) Products. Forensic Sci. Int. 299, 195–202. 10.1016/j.forsciint.2019.04.009 31059866

[B15] Franz B. Dhanani L. Y. Miller W. C. (2021). Rural-urban Differences in Physician Bias toward Patients with Opioid Use Disorder. Psychiatr. Serv. 72 (8), 874–879. 10.1176/appi.ps.202000529 33622043

[B16] Garcia-Romeu A. Cox D. J. Smith K. E. Dunn K. E. Griffiths R. R. (2020). Kratom (Mitragyna Speciosa): User Demographics, Use Patterns, and Implications for the Opioid Epidemic. Drug Alcohol Depend 208, 107849. 10.1016/j.drugalcdep.2020.107849 32029298 PMC7423016

[B17] Griffin O. H. Daniels J. A. Gardner E. A. (2016). Do You Get what You Paid for? an Examination of Products Advertised as Kratom. J. Psychoactive Drugs 48 (5), 330–335. 10.1080/02791072.2016.1229876 27669103

[B18] Grundmann O. (2017). Patterns of Kratom Use and Health Impact in the US-Results from an Online Survey. Drug Alcohol Depend 176, 63–70. 10.1016/j.drugalcdep.2017.03.007 28521200

[B53] Grundmann O. Babin J. K. Henningfield J. E. Garcia-Romeu A. Kruegel A. C. Prozialeck W. C. (2021). Kratom Use in the United States: A Diverse and Complex Profile. Addiction 116 (1), 202–203. 10.1111/add.15173 32602213 PMC7772230

[B19] Havens J. R. Oser C. B. Leukefeld C. G. Webster J. M. Martin S. S. O'Connell D. J. (2007). Differences in Prevalence of Prescription Opiate Misuse Among Rural and Urban Probationers. Am. J. Drug Alcohol. Abuse 33 (2), 309–317. 10.1080/00952990601175078 17497554

[B20] Havens J. R. Young A. M. Havens C. E. (2011). Nonmedical Prescription Drug Use in a Nationally Representative Sample of Adolescents: Evidence of Greater Use Among Rural Adolescents. Arch. Pediatr. Adolesc. Med. 165 (3), 250–255. 10.1001/archpediatrics.2010.217 21041587

[B21] Hedegaard H. Spencer M. R. (2021). Urban-Rural Differences in Drug Overdose Death Rates, 1999-2019. Hyattsville, MD: US Department of Health and Human Services, Centers for Disease Control and Prevention, National Center for Health Statistics.

[B22] Hiranita T. Leon F. Felix J. S. Restrepo L. F. Reeves M. E. Pennington A. E. (2019). The Effects of Mitragynine and Morphine on Schedule-Controlled Responding and Antinociception in Rats. Psychopharmacology (Berl) 236 (9), 2725–2734. 10.1007/s00213-019-05247-7 31098655 PMC6697625

[B23] Jacobson N. Horst J. Wilcox-Warren L. Toy A. Knudsen H. K. Brown R. (2020). Organizational Facilitators and Barriers to Medication for Opioid Use Disorder Capacity Expansion and Use. J. Behav. Health Serv. Res. 47 (4), 439–448. 10.1007/s11414-020-09706-4 32347426 PMC7578054

[B24] Jansen K. L. Prast C. J. (1988). Ethnopharmacology of Kratom and the Mitragyna Alkaloids. J. Ethnopharmacol 23 (1), 115–119. 10.1016/0378-8741(88)90121-3 3419199

[B25] Jarvey N. (2020). The Joe Rogan Experience’ Is Spotify’s Most Popular Podcast. Billboard. Available at https://www.billboard.com/articles/news/9491714/joe-rogan-experience-spotify-most-popular-podcast/.

[B26] Jones E. B. (2018). Medication-Assisted Opioid Treatment Prescribers in Federally Qualified Health Centers: Capacity Lags in Rural Areas. J. Rural Health 34 (1), 14–22. 10.1111/jrh.12260 28842930

[B28] Kruegel A. C. Grundmann O. (2018). The Medicinal Chemistry and Neuropharmacology of Kratom: A Preliminary Discussion of a Promising Medicinal Plant and Analysis of its Potential for Abuse. Neuropharmacology 134, 108–120. 10.1016/j.neuropharm.2017.08.026 28830758

[B29] Kruegel A. C. Uprety R. Grinnell S. G. Langreck C. Pekarskaya E. A. Le Rouzic V. (2019). 7-Hydroxymitragynine Is an Active Metabolite of Mitragynine and a Key Mediator of its Analgesic Effects. ACS Cent. Sci. 5 (6), 992–1001. 10.1021/acscentsci.9b00141 31263758 PMC6598159

[B30] Lister J. J. Weaver A. Ellis J. D. Himle J. A. Ledgerwood D. M. (2020). A Systematic Review of Rural-specific Barriers to Medication Treatment for Opioid Use Disorder in the United States. Am. J. Drug Alcohol. Abuse 46 (3), 273–288. 10.1080/00952990.2019.1694536 31809217

[B31] Lofquist D. Lugaila T. O'Connell M. Feliz S. (2012). Households and Families: 2010 (C2010BR-14). Suitland, MD: US Department of Commerce, US Census Bureau.

[B32] Luu H. Slavova S. Freeman P. R. Lofwall M. Browning S. Bush H. (2019). Trends and Patterns of Opioid Analgesic Prescribing: Regional and Rural-Urban Variations in Kentucky from 2012 to 2015. J. Rural Health 35 (1), 97–107. 10.1111/jrh.12300 29664203

[B33] Mack K. A. Jones C. M. Ballesteros M. F. (2017). Illicit Drug Use, Illicit Drug Use Disorders, and Drug Overdose Deaths in Metropolitan and Nonmetropolitan Areas - United States. MMWR Surveill. Summ. 66 (19), 1–12. 10.15585/mmwr.ss6619a1 PMC5829955 29049278

[B34] Media Monitors (2021). Audience Demographic Variations Are Specific to Genre and Even Individual Podcasts. Available at https://www.mediamonitors.com/audience-demographic-variations-specific-to-genre/.

[B35] Meit M. Heffernan M. Tanenbaum E. Hoffmann T. (2017). Appalachian Diseases of Despair. Washington DC: Appalachian Regional Commission.

[B37] Miller J. D. Crowe M. Weiss B. Maples-Keller J. L. Lynam D. R. (2017). Using Online, Crowdsourcing Platforms for Data Collection in Personality Disorder Research: The Example of Amazon's Mechanical Turk. Personal. Disord. 8 (1), 26–34. 10.1037/per0000191 28045305

[B39] Monnat S. M. (2020). “Opioid Crisis in the Rural U.S,” in Rural Families and Communities in the United States. Editors Glick J. E. McHale S. M. King V. (Springer International Publishing), 10, 117–143. 10.1007/978-3-030-37689-5_5

[B40] Mortensen K. Hughes T. L. (2018). Comparing Amazon's Mechanical Turk Platform to Conventional Data Collection Methods in the Health and Medical Research Literature. J. Gen. Intern. Med. 33 (4), 533–538. 10.1007/s11606-017-4246-0 29302882 PMC5880761

[B41] Mosher H. Zhou Y. Thurman A. L. Sarrazin M. V. Ohl M. E. (2017). Trends in Hospitalization for Opioid Overdose Among Rural Compared to Urban Residents of the United States, 2007-2014. J. Hosp. Med. 12 (11), 925–929. 10.12788/jhm.2793 29091981

[B42] Mun M. Wong A. (2020). Kratom and Phenibut: A Concise Review for Psychiatric Trainees. Am. J. Psychiatry Residents' J. 16 (2), 6–8. 10.1176/appi.ajp-rj.2020.160203

[B43] Ng J. Y. Zhang C. J. Ahmed S. (2021). Dietary and Herbal Supplements for Fatigue: A Quality Assessment of Online Consumer Health Information. Integr. Med. Res. 10 (4), 100749. 10.1016/j.imr.2021.100749 34141579 PMC8187245

[B44] Nicewonder J. A. Buros A. F. Veltri C. A. Grundmann O. (2019). Distinct Kratom User Populations across the United States: A Regional Analysis Based on an Online Survey. Hum. Psychopharmacol. 34 (5), e2709. 10.1002/hup.2709 31347212

[B45] Nolte K. Drew A. L. Friedmann P. D. Romo E. Kinney L. M. Stopka T. J. (2020). Opioid Initiation and Injection Transition in Rural Northern New England: A Mixed-Methods Approach. Drug Alcohol Depend 217, 108256. 10.1016/j.drugalcdep.2020.108256 32947174 PMC7769168

[B46] Obeng S. Wilkerson J. L. León F. Reeves M. E. Restrepo L. F. Gamez-Jimenez L. R. (2021). Pharmacological Comparison of Mitragynine and 7-Hydroxymitragynine: *In Vitro* Affinity and Efficacy for μ-Opioid Receptor and Opioid-like Behavioral Effects in Rats. J. Pharmacol. Exp. Ther. 376 (3), 410–427. 10.1124/jpet.120.000189 33384303 PMC7923387

[B38] Palamar J. J. (2021). Past-Year Kratom Use in the US: Estimates From a Nationally Representative Sample. Am. J. Prev. Med. 10.1016/j.amepre.2021.02.004 PMC8319032 34027890

[B47] Paulozzi L. J. Xi Y. (2008). Recent Changes in Drug Poisoning Mortality in the United States by Urban-Rural Status and by Drug Type. Pharmacoepidemiol. Drug Saf. 17 (10), 997–1005. 10.1002/pds.1626 18512264

[B48] Peer E. Vosgerau J. Acquisti A. (2014). Reputation as a Sufficient Condition for Data Quality on Amazon Mechanical Turk. Behav. Res. Methods 46 (4), 1023–1031. 10.3758/s13428-013-0434-y 24356996

[B49] Prevete E. Hupli A. Marrinan S. Singh D. Udine B. D. Bersani G. (2021). Exploring the Use of Kratom (Mitragyna Speciosa) via the YouTube Data Tool: A Novel Netnographic Analysis. Emerging Trends Drugs Addictions, Health 1, 100007. 10.1016/j.etdah.2021.100007

[B50] Prunuske J. P. St Hill C. A. Hager K. D. Lemieux A. M. Swanoski M. T. Anderson G. W. (2014). Opioid Prescribing Patterns for Non-malignant Chronic Pain for Rural versus Non-rural US Adults: A Population-Based Study Using 2010 NAMCS Data. BMC Health Serv. Res. 14 (1), 563. 10.1186/s12913-014-0563-8 25407745 PMC4241226

[B51] Rogan J. Host (2018). #1136 - Hamilton Morris (No. 1136) [Video Podcast Episode]. In the Joe Rogan Experience. Spotify. Available at: https://open.spotify.com/episode/4xBgDDb5rBrTWo0qIUXfuG.

[B52] Rogan J. Host (2019). #1296 – Joe List (No. 1296) [Video Podcast Episode]. In the Joe Rogan Experience. Spotify. Available at: https://open.spotify.com/episode/3lKc4j1QoHt5qcxDbgO54p.

[B27] Schimmel J. Amioka E. Rockhill K. Haynes C. M. Black J. C. Dart R. C. (2021). Prevalence and Description of Kratom (Mitragyna speciosa) Use in the United States: A Cross-Sectional Study. Addiction 116, 176–181. 10.1111/add.15082 32285981

[B54] Schnell M. Gupta S. Powell D. M. Bradford W. D. Simon K. (2020). “County-Level Effects of the Introduction of Abuse-Deterrent Opioids: Can the Reformulation of Oxycontin Explain the Urban-Rural Divide in the Prevalence of the Substance Use Epidemic,” in 9th Annual Conference of the American Society of Health Economists, St. Louis, Missouri (ASHECON).

[B55] Sexton R. L. Carlson R. G. Leukefeld C. G. Booth B. M. (2008). Barriers to Formal Drug Abuse Treatment in the Rural South: A Preliminary Ethnographic Assessment. J. Psychoactive Drugs 40 (2), 121–129. 10.1080/02791072.2008.10400621 18720660

[B56] Smith K. E. Lawson T. (2017). Prevalence and Motivations for Kratom Use in a Sample of Substance Users Enrolled in a Residential Treatment Program. Drug Alcohol Depend 180, 340–348. 10.1016/j.drugalcdep.2017.08.034 28950240

[B57] Smith K. E. Rogers J. M. Schriefer D. Grundmann O. (2021b). Therapeutic Benefit with Caveats? Analyzing Social media Data to Understand the Complexities of Kratom Use. Drug Alcohol Depend 226, 108879. 10.1016/j.drugalcdep.2021.108879 34216869 PMC8355181

[B58] Smith K. E. Rogers J. M. Strickland J. C. Epstein D. H. (2021a). When an Obscurity Becomes Trend: Social-media Descriptions of Tianeptine Use and Associated Atypical Drug Use. Am. J. Drug Alcohol. Abuse 47, 455–466. 10.1080/00952990.2021.1904408 33909525 PMC8380661

[B59] Smith K. E. Rogers J. M. Dunn K. E. Schriefer D. Grundmann O. McCurdy C. (2021d). Searching for a Signal: Self-Reported Kratom Dose-Effect Relationship Among a US Sample. Front. Pharmacol.. Submitted. 10.3389/fphar.2022.765917 PMC8921773 35300296

[B60] Smith K. E. Rogers J. M. Schriefer D. Grundmann O. Rogers J. M. Swogger M. T. (2021c). Therapeutic Benefit with Caveats? Analyzing Social media Data to Understand the Complexities of Kratom Use. Drug and Alcohol Dependence 226, 108879. 10.1016/j.drugalcdep.2021.108879 34216869 PMC8355181

[B61] Snell-Rood C. Carpenter-Song E. (2018). Depression in a Depressed Area: Deservingness, Mental Illness, and Treatment in the Contemporary Rural U.S. Soc. Sci. Med. 219, 78–86. 10.1016/j.socscimed.2018.10.012 30391873 PMC6290352

[B62] Strickland J. C. Stoops W. W. Dunn K. E. Smith K. E. Havens J. R. (2021). The Continued Rise of Methamphetamine Use Among People Who Use Heroin in the United States. Drug Alcohol Depend 225, 108750. 10.1016/j.drugalcdep.2021.108750 34052690 PMC8282713

[B63] Strickland J. C. Stoops W. W. (2018). Feasibility, Acceptability, and Validity of Crowdsourcing for Collecting Longitudinal Alcohol Use Data. J. Exp. Anal. Behav. 110 (1), 136–153. 10.1002/jeab.445 29873806

[B64] Strickland J. C. Stoops W. W. (2019). The Use of Crowdsourcing in Addiction Science Research: Amazon Mechanical Turk. Exp. Clin. Psychopharmacol. 27 (1), 1–18. 10.1037/pha0000235 30489114

[B65] Swogger M. T. Walsh Z. (2018). Kratom Use and Mental Health: A Systematic Review. Drug Alcohol Depend 183, 134–140. 10.1016/j.drugalcdep.2017.10.012 29248691

[B66] Thomas N. van de Ven K. Mulrooney K. J. D. (2020). The Impact of Rurality on Opioid-Related Harms: A Systematic Review of Qualitative Research. Int. J. Drug Pol. 85, 102607. 10.1016/j.drugpo.2019.11.015 31864787

[B67] Todd D. A. Kellogg J. J. Wallace E. D. Khin M. Flores-Bocanegra L. Tanna R. S. (2020). Chemical Composition and Biological Effects of Kratom (Mitragyna Speciosa): *In Vitro* Studies with Implications for Efficacy and Drug Interactions. Sci. Rep. 10 (1), 19158. 10.1038/s41598-020-76119-w 33154449 PMC7645423

[B68] Woolf S. H. Schoomaker H. Hill L. Orndahl C. M. (2019). The Social Determinants of Health and the Decline in US Life Expectancy: Implications for Appalachia. J. Appalachian Health 1 (1), 2. 10.13023/jah.0101.02 PMC9173599 35769540

[B69] Yarkoni T. (2020). The Generalizability Crisis. Behav. Brain Sci., 1–37. 10.1017/S0140525X20001685 33342451 PMC10681374

